# Apert’s Syndrome: Report of a New Case and its Management

**DOI:** 10.5005/jp-journals-10005-1009

**Published:** 2008-12-26

**Authors:** Shweta Dixit, Asha Singh, Mamatha GS, Rajiv S Desai, Prashant Jaju

**Affiliations:** 1 Postgraduate Student, Department of Pedodontics and Preventive Dentistry, Dr DY Patil Dental College and Hospital Pune, Maharashtra, India; 2 Professor and Director of Postgraduate Studies, Department of Pedodontics and Preventive Dentistry, Dr DY Patil Dental College and Hospital, Pune, Maharashtra, India; 3 Postgraduate Student, Department of Oral and Maxillofacial Pathology, Dr DY Patil Dental College and Hospital Pune, Maharashtra, India; 4 Professor, Department of Oral and Maxillofacial Pathology, Dr DY Patil Dental College and Hospital, Pune, Maharashtra, India; 5 Postgraduate Student, Department of Oral Medicine and Radiology, Dr DY Patil Dental College and Hospital, Pune, Maharashtra, India

## Abstract

In this article, an interesting case of Apert syndrome in a 14-year-old boy with characteristic craniosynostosis, acrocephaly, midface hypoplasia, pharyngeal attenuation, ocular manifestations, and syndactyly of the hands and feet is presented. The case is discussed in the light of relevant literature. A precise clinical differentiation must be made since considerable overlap of the features of various other syndromes could give rise to difficulties in diagnosing this condition. Besides detection and timely recognition of the syndrome to allow adequate dental care, screening at periodic intervals is merited to improve the overall quality of life of these patients.

**Clinical relevance**

1. This paper highlights the importance of the dentist as well as the specialist in the recognition and oral care of children with this syndrome.
2. Children with teeth of unusual anatomy present a challenge for conventional dentistry.
3. It is important for a pedodontist to evaluate and intervene the malrelationship of the jaws to reduce the complexity of further orthodontic treatment.

**Objectives statement:** The reader should understand the clinical implications of recognition of this syndrome and provision of early treatment, with a purpose to reducing the duration and complexity of further treatment.

## INTRODUCTION

Apert syndrome (acrocephalosyndactyly) is a rare condition, occurring in about 1 in every 1,00,000 to 1,60,000 live births characterized by craniosynostosis, acrocephaly and syndactyly of the hands and feet, often combined with anomalies of other organs.^1^ Though this syndrome was mentioned as early as 1842 by Baumgartner, the eponymic credit was given to Dr Eugene Apert for presentation of the syndrome in 1906. It can be inherited as an autosomal dominant trait, or may develop as a spontaneous mutation often associated with increased paternal age. More than 98% of cases with Apert’s syndrome are caused by specific missense substitution mutations involving adjacent amino acids (Ser252Trp, Ser252Phe, Pro253Arg) in the linker between the second and third extracellular immunoglobulin domain of Fibroblast Growth Factor Receptor 2 (FGFR2), which maps to chromosome bands 10q25-q26. The remaining cases are due to mutations in or near exon 9 of FGFR2.^2^

## CASE REPORT

**Fig. 1.**
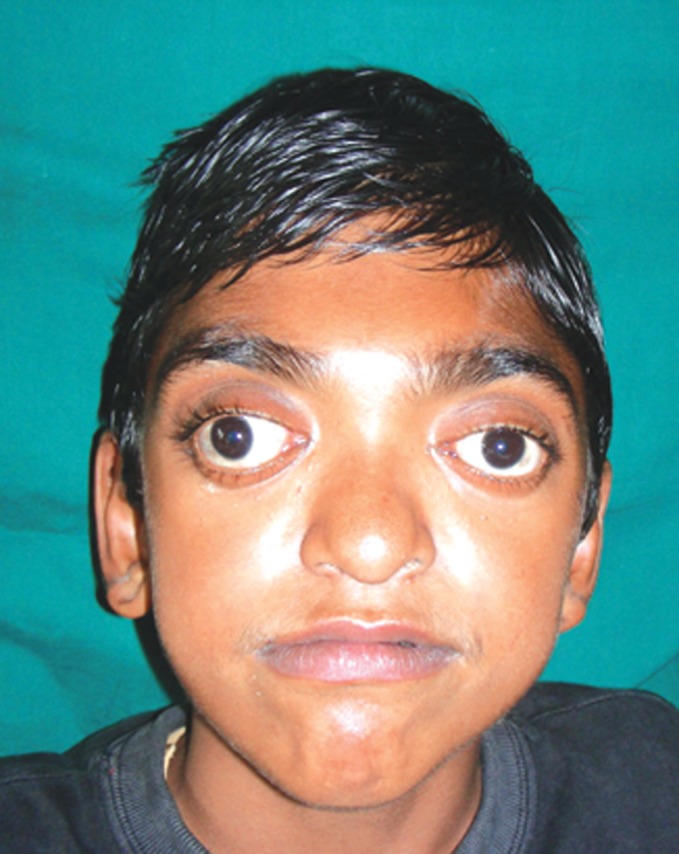
Extraoral photograph showing strabismus and hypertelorism

**Fig. 2.**
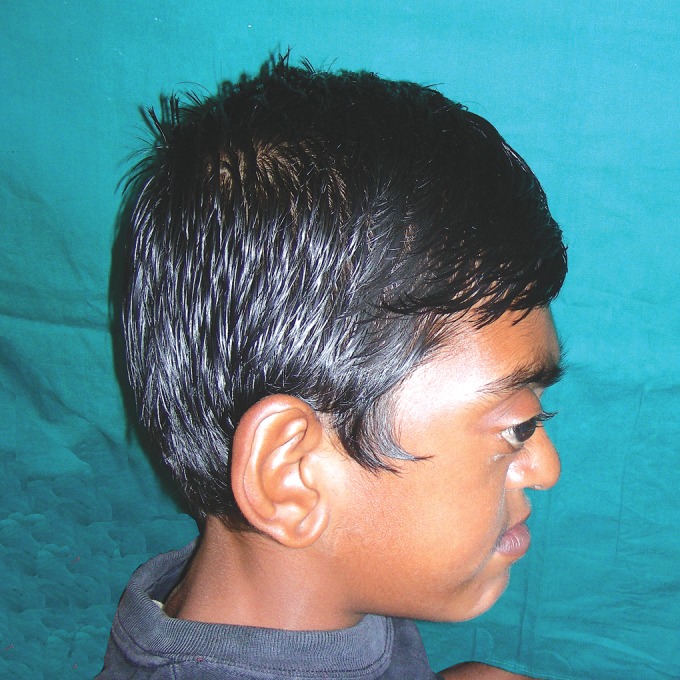
Extraoral photograph showing midface hypoplasia with a relative mandibular prognathism and depressed nasal bridge

**Fig. 3.**
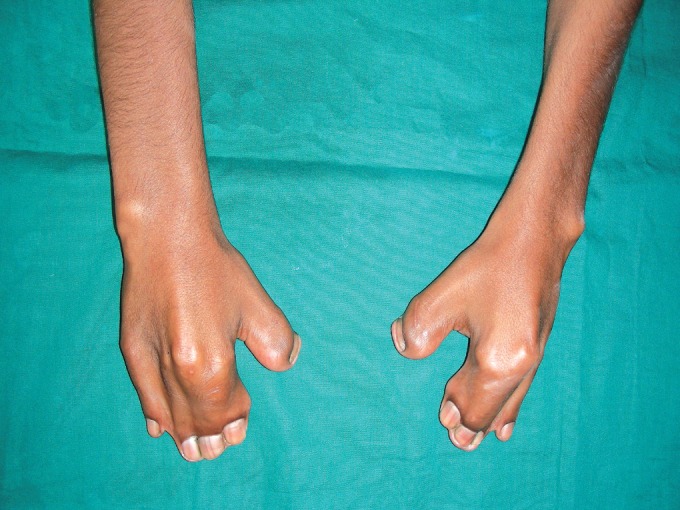
Both hands showing syndactyly of 2nd , 3rd and 4th digits

**Fig. 4.**
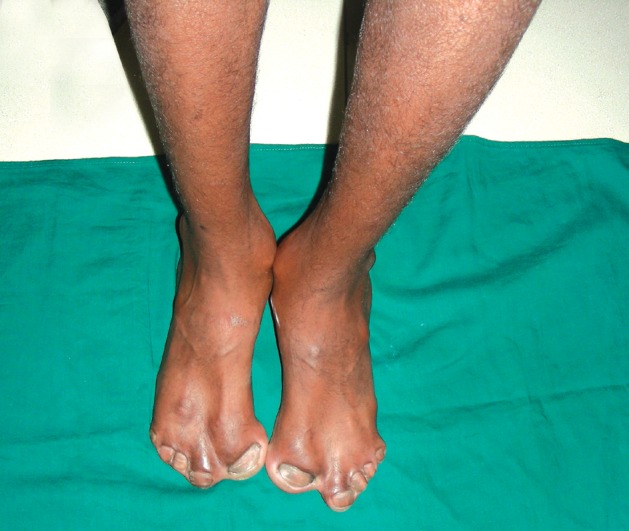
Both feet showing syndactyly of all digits

**Fig. 5.**
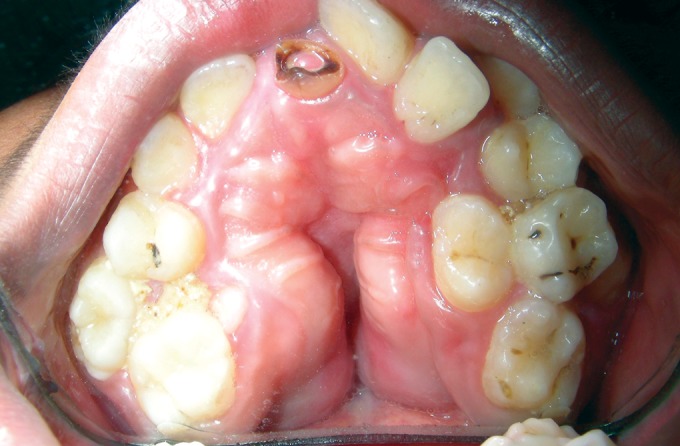
Intraoral photograph showing high arched palate with pseudocleft, ectopic teeth eruption, crowding of teeth and malformed molars

**Fig. 6.**
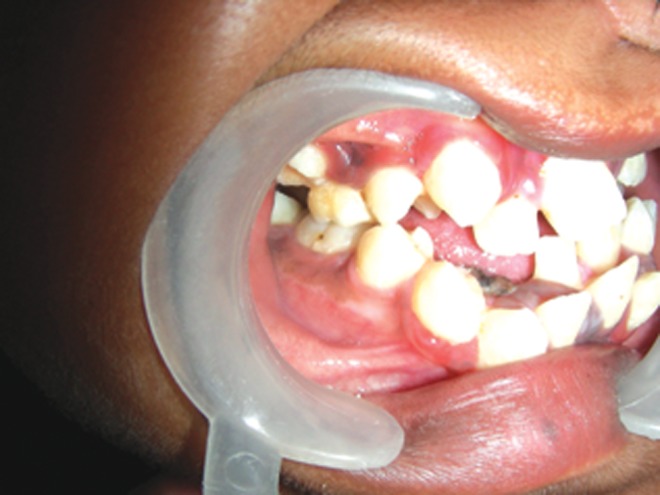
Intraoral photograph showing macrodontia of premolars, crowding of teeth and class III malocclusion

A 14-year-old boy in good general health was referred to the Department of Pedodontics and Preventive Dentistry for orthodontic evaluation. The patient presented with unusual craniofacial and dental features, which prompted a further detailed examination of the case. He was the second child of an apparently normal mother. No history of a consanguineous marriage of his parents was present. Detailed family history showed that patient’s parents and his sibling, a 21-year-old brother did not manifest any related findings. The patient had normal developmental milestones with minimal signs of mental retardation. Speech was slightly incoherent. Patient had abnormal facial features evident as acrocephaly, brachycephaly, flat occiput and high prominent forehead. Clinical examination revealed strabismus, ocular proptosis, hypertelorism, midface hypoplasia with a relative mandibular prognathism and depressed nasal bridge (Figs 1 and 2). Characteristic limb defects in the form of syndactyly of the hands included complete fusion of 2nd , 3rd and 4th digits along with complete fusion of all digits of the feet (Figs 3 and 4). However, the patient has adequate manual dexterity to be able to write legibly. Intraoral examination revealed hypoplastic maxilla, high arched palate with pseudocleft, ectopic teeth eruption with concomitant severe dental crowding and carious teeth, class III malocclusion, macrodontia of premolars, malformed molars, over-retained deciduous maxillary left central incisor and mandibular canine (Figs 5 and 6).

**Fig. 7.**
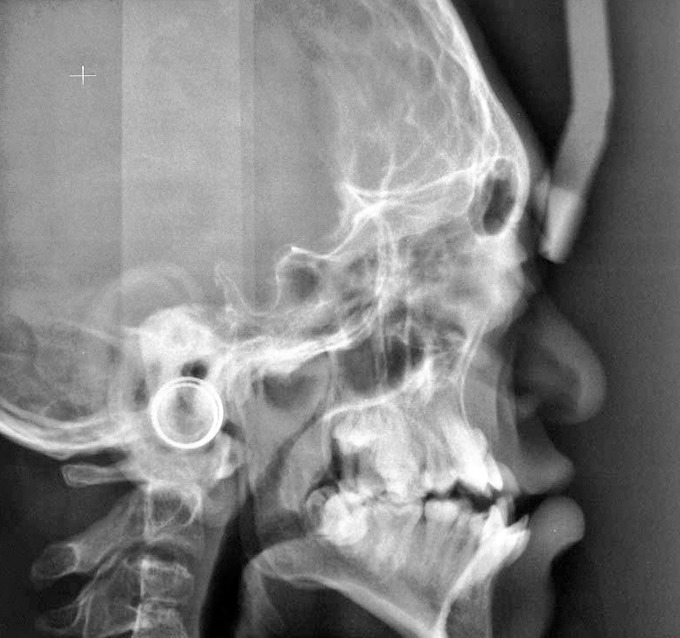
Skull radiograph revealing brachycephaly with characteristic beaten metal appearance

**Fig. 8.**
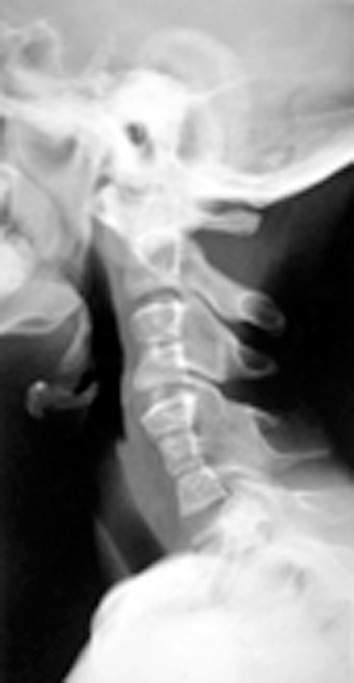
Cervical spine X-ray showing congenital fusion of neural arches of C3-C4 and C5-C6-C7

Radiographic examination of skull showed brachycephaly with characteristic beaten metal appearance (Fig. 7). Lateral X-ray view of cervical spine showed congenital fusion of neural arches of C3-C4 and C5-C6- C7, small sized vertebral bodies from C3-C7 and rudimentary discs between C3-C4, C5-C6 and C6-C7 (Fig. 8).

**Fig. 9.**
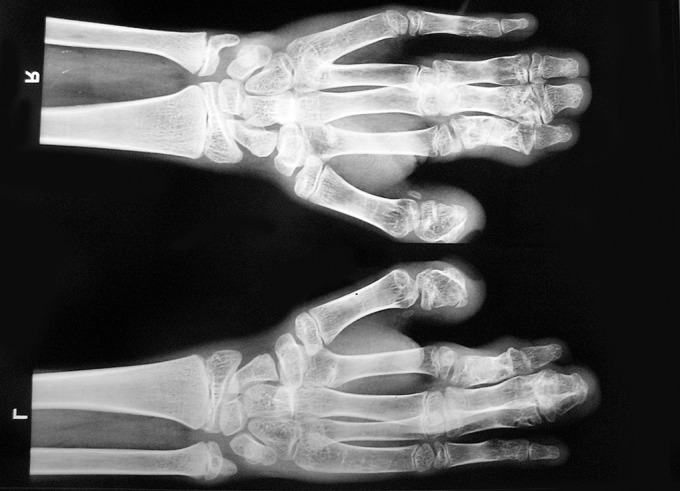
Hands X-ray showing syndactyly of 2nd 3rd and 4th digits

**Fig. 10.**
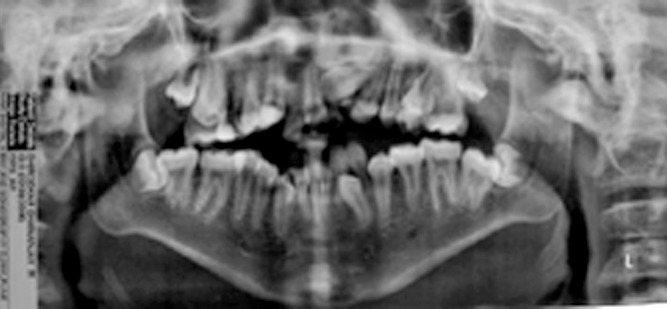
Panoramic radiograph showing over retained teeth impacted teeth, macrodontia of premolars and prominent gonial angle with vertical ramus of the mandible.

**Fig. 11.**
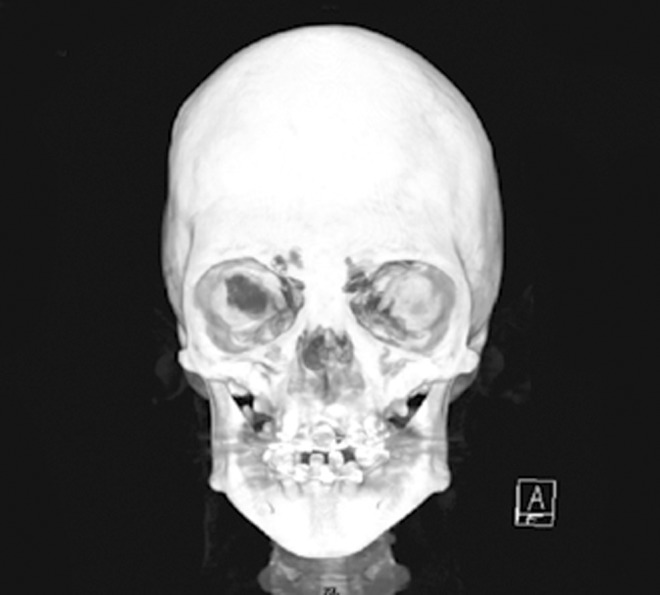
Three dimensional computerized tomography showing craniostenosis

Anteroposterior X-ray view of both hands showed syndactyly of index, middle and ring fingers (Fig. 9). Dosoplantar X-ray view of both feet showed syndactyly of all the toes. Panoramic radiograph showed crowding, over retained and impacted teeth, macrodontia of premolars and prominent gonial angle with prominent vertical ramus of the mandible (Fig. 10). Three dimensional computerized tomography revealed craniostenosis (Fig. 11).

## DIAGNOSIS

The final diagnosis of Apert syndrome (acrocephalosyndactyly) was made on the basis of typical craniofacial features after ruling out similar syndromes like Carpenter syndrome, Pfeiffer syndrome, Beare-Stevenson syndrome and Crouzon syndrome. An orthodontic diagnosis of Angle’s Class III tendency with a vertical growth pattern was made with SNA being 88° and SNB being 73°. ANB was found to be 15°. Since these values seemed to be misleading, McNamara’s analysis was done. Both maxilla and mandible were found to be retrognathic to the cranial base.

**Fig. 12.**
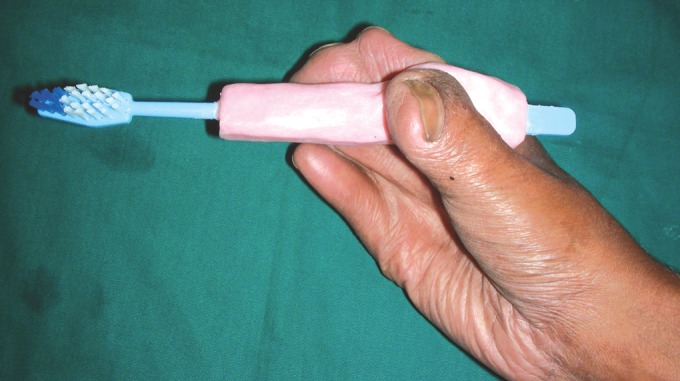
Patient holding the custom made toothbrush given to improve manual dexterity

## MANAGEMENT

**Fig. 13.**
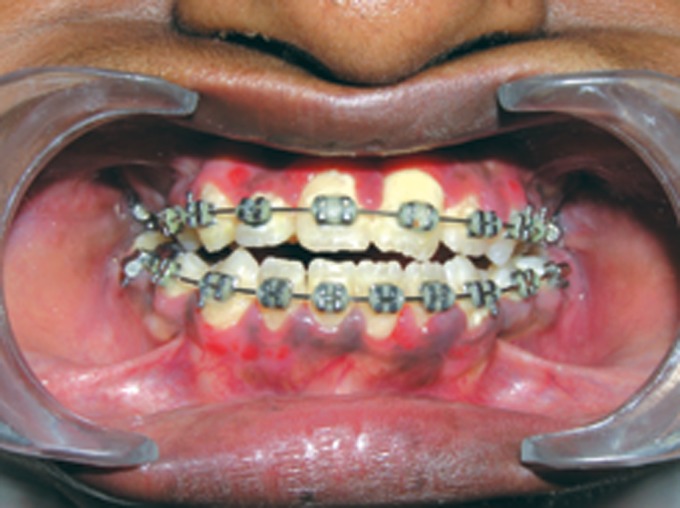
Frontal view of the arches at present

**Fig. 14.**
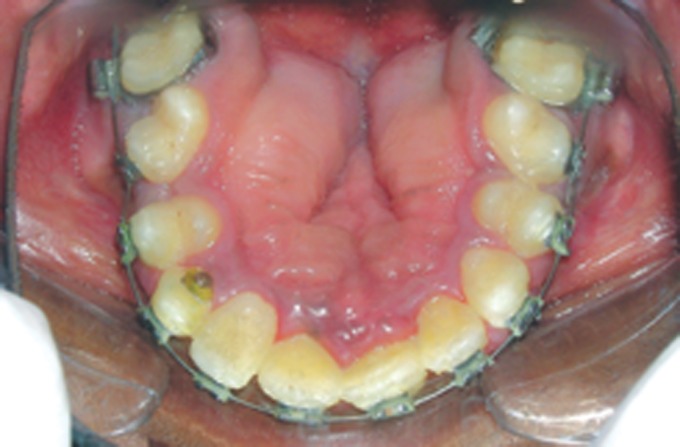
Current status of the maxillary arch

**Fig. 15.**
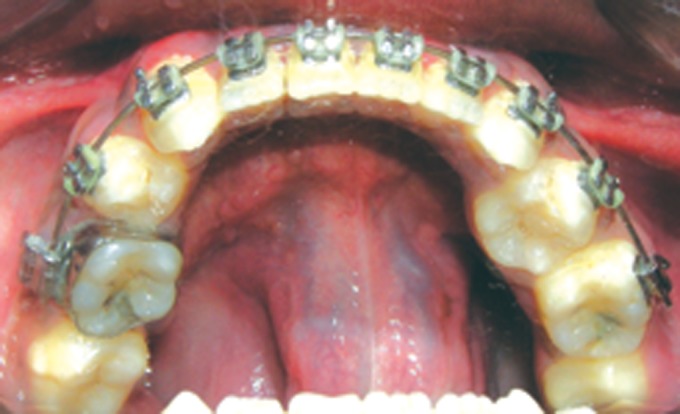
Current status of the mandibular arch

A custom made toothbrush was fabricated for the patient using his finger prints as a template to improve manual dexterity for oral hygiene (Fig. 12). The following teeth were extracted due to severe crowding and non-availability of spacespaceleft and right maxillary first molars, left deciduous maxillary second molar, right deciduous maxillary central incisor, left deciduous mandibular first molar and right and left mandibular first premolars. A trihelix was luted onto the permanent maxillary second molars for maxillary expansion (Fig. 13). Six months treatment provided an increase in maxillary width but did not entirely eliminate the pseudocleft, although the bunching of tissue in the palate appeared less prominent. Fixed orthodontic treatment was initiated using 0.014 CuNiTi followed by 0.016 NiTi for alignment and levelling of the maxillary arch (Fig. 14). Molar derotation was done to create space for alignment of the premolars. Surgical exposure of the left central incisor was done under local anesthesia. Incisor was pulled occlusally using elastics. For the mandibular arch, initial alignment and levelling were done using the same round wires as for the maxillary arch, followed by 16 × 16 and 16 × 20 rectangular NiTi wires. 0.016 stainless steel AJ Wilcock was used for canine retraction to close the extraction spaces (Fig. 15). Further treatment includes planned Le Fort III osteotomy to reduce the midface deficiency. Plastic surgery for separation of the digits of the hands may be a difficult proposition due to bony fusion. Regular speech therapy sessions are being undertaken to improve the clarity of his speech.

## DISCUSSION

The two most common of the syndromic craniosynostoses, collectively known as acrocephalosyndactyly, are Crouzon’s and Apert’s, which together make up 70% of such cases.^3^ As described by Dr Eugene Apert in 1906, Apert syndrome is characterized by the clinical triad of craniosynostosis, midface hypoplasia, and symmetric syndactyly of the hands and feet. The inheritance of Apert’s syndrome is autosomal dominant but most cases arise as spontaneous mutations that appear to originate almost exclusively in the paternal germ line.^1^,^4^ Two mutations found in adjacent codons have been identified as being responsible for the defects seen in Apert syndrome. Leading to altered structure in the Fibroblast Growth Factor Receptor (FGFR) 2, the two mutations on chromosome 10q are 755C->G, resulting in a Ser252 to Trp change, which is found in about two-thirds of patients, and 758C->G, resulting in a Pro253 to Arg change, seen in the remaining one third patients.^1^ Suture progenitor cells with Fibroblast Growth Factor Receptors (FGFR) 2 that have undergone a mutation cannot transduce signals from the extracellular Fibroblast Growth Factors (FGF). Therefore, these cells do not receive the signal to produce the necessary fibrous material essential for a normal calvarial suture.^4^

Orofacial manifestations of Apert syndrome include flat facies, shallow orbits, hypertelorism, hypoplastic maxilla and high arched narrow palate with or without bifid uvula.^2^,^5^ Other associated anomalies that have been observed are pyloric stenosis, ectopic anus, pulmonary aplasia, pulmonary arterial and valve abnormality, heart defects, polycystic kidneys and bicornuate uterus.^1^ Our case has all the classical clinical and radiological features along with malformed premolars and molars and prominent gonial angle with vertical ramus of the mandible which has not been mentioned so far in the literature.

## CONCLUSION

The necessity for a dentist to be capable of recognizing and dealing with genetic diseases is becoming increasingly important due to the number of recognized genetic traits and diseases that involve orofacial structures. A multidisciplinary approach is necessary due to the complex nature of this syndrome. A craniofacial team may consist of a craniofacial surgeon, neurosurgeon, neurologist, ENT, audiologist, speech pathologist, pedodontist, oral surgeon, ophthalmologist, psychologist and orthodontist. The team approach is essential to determine the best collaborative corrective plan for the deficiencies of each individual child according to the combination of features displayed. Treatment includes conventional surgical advancement (Le Fort III osteotomy) of the midface although there are numerous reports which mention that gradual advancement of the midface region using osteogenic distraction (Ilizarov procedure) may provide a more stable correction. Early optimization of hearing with possible hearing aids, airway management, psychological counseling, speech correction and genetic counseling are required for a comprehensive rehabilitation of the child in society. When diagnosed early, initial surgery for craniosynostosis performed as early as 3 months of age can result in significant cosmetic improvement. Facial cosmetic reconstruction can be undertaken later for dysmorphisms. Shunting procedure for reduction of intracranial pressure can be undertaken. Surgical separation of digits has been found to provide relatively little functional improvement. Fortunately, in the majority of cases, no special dietary recommendations and no restriction of activity is required.^6^

Thus, in its full-blown clinical form, Apert syndrome is easily diagnosed and the oral and dental signs are so peculiar that they constitute a fundamental cue for the differential diagnosis and genetic counseling. Treatment involves a stepwise approach tailor-made to the specific needs of the patient based on his unique combination of features.

## References

[1] Chonka ZK, Beall DP, Jennings BT, Wu DH, Ly JQ, Wolff JD (2004). Apert's syndrome. Appl Radiol.

[2] Acharya AB, Sivapathasundharam B, Rajendran R, Sivapathasundharam B (2006). Forensic odontology. Shafer's textbook of oral pathology.

[3] Cohen MM Jr. (1988). Craniosynostosis update 1987. Am J Med Genet Suppl.

[4] Batra P, Duggal R, Prakash H (2002). Dentofacial characteristics in
Apert syndrome: a case report. J Indian Soc Pedod Prev Dent.

[5] Neville BW, Damm DD, Allen CM, Bouquot JE (2002). Oral and maxillofacial pathology.

[6] Cohen MM Jr. (1986). Craniosynostosis diagnosis, evaluation, and management.

